# Linking Short-Chain N-Acyl Homoserine Lactone-Mediated Quorum Sensing and Replant Disease: A Case Study of *Rehmannia glutinosa*

**DOI:** 10.3389/fpls.2020.00787

**Published:** 2020-06-17

**Authors:** Qian Li, Yanhong Wu, Juanying Wang, Bo Yang, Jun Chen, Hongmiao Wu, Zhongyi Zhang, Cuihong Lu, Wenxiong Lin, Linkun Wu

**Affiliations:** ^1^College of Life Sciences, Fujian Agriculture and Forestry University, Fuzhou, China; ^2^Key Laboratory of Crop Ecology and Molecular Physiology, Fujian Agriculture and Forestry University, Fuzhou, China; ^3^College of Crop Science, Fujian Agriculture and Forestry University, Fuzhou, China; ^4^Wenxian Institute of Agricultural Sciences, Jiaozuo, China; ^5^Fujian Provincial Key Laboratory of Agroecological Processing and Safety Monitoring, Fujian Agriculture and Forestry University, Fuzhou, China

**Keywords:** *Rehmannia glutinosa*, replant disease, quorum sensing, root exudate, quorum quenching

## Abstract

*Rehmannia glutinosa*, a perennial medicinal plant, suffers from severe replant disease under consecutive monoculture. The rhizosphere microbiome is vital for soil suppressiveness to diseases and for plant health. Moreover, N-acyl homoserine lactone (AHL)-mediated quorum sensing (QS) regulates diverse behavior in rhizosphere-inhabiting and plant pathogenic bacteria. The dynamics of short-chain AHL-mediated QS bacteria driven by consecutive monoculture and its relationships with *R. glutinosa* replant disease were explored in this study. The screening of QS bacteria showed that 65 out of 200 strains (32.5%) randomly selected from newly planted soil of *R. glutinosa* were detected as QS bacteria, mainly consisting of *Pseudomonas* spp. (55.4%). By contrast, 34 out of 200 (17%) strains from the diseased replant soil were detected as QS bacteria, mainly consisting of Enterobacteriaceae (73.5%). Functional analysis showed most of the QS bacteria belonging to the *Pseudomonas* genus showed strong antagonistic activities against *Fusarium oxysporum* or *Aspergillus flavus*, two main causal agents of *R. glutinosa* root rot disease. However, the QS strains dominant in the replant soil caused severe wilt disease in the tissue culture seedlings of *R. glutinosa*. Microbial growth assays demonstrated a concentration-dependent inhibitory effect on the growth of beneficial QS bacteria (i.e., *Pseudomonas brassicacearum*) by a phenolic acid mixture identified in the root exudates of *R. glutinosa*, but the opposite was true for harmful QS bacteria (i.e., *Enterobacter* spp.). Furthermore, it was found that the population of quorum quenching (QQ) bacteria that could disrupt the beneficial *P. brassicacearum* SZ50 QS system was significantly higher in the replant soil than in the newly planted soil. Most of these QQ bacteria in the replant soil were detected as *Acinetobacter* spp. The growth of specific QQ bacteria could be promoted by a phenolic acid mixture at a ratio similar to that found in the *R. glutinosa* rhizosphere. Moreover, these quorum-quenching bacteria showed strong pathogenicity toward the tissue culture seedlings of *R. glutinosa.* In conclusion, consecutive monoculture of *R. glutinosa* contributed to the imbalance between beneficial and harmful short-chain AHL-mediated QS bacteria in the rhizosphere, which was mediated not only by specific root exudates but also by the QQ bacterial community.

## Introduction

To ensure food security and meet market needs, the practice of consecutive monoculture is becoming popular in intensive agriculture. However, large-scale crop monoculture results in many problems including a loss of crop genetic diversity, an increase in disease incidence, a decline in crop quality and even the fragility of ecosystem functioning (Jacques and Jacques, 2012; Mariotte et al., 2018; Li et al., 2019). Consecutive monoculture problems, also known as replant problems or replant disease, are especially severe in the cultivation of medicinal herbs, such as *Rehmannia glutinosa*, *Panax notoginseng*, *Pseudostellaria heterophylla*, and *Panax ginseng* (Dong et al., 2018a; Luo et al., 2019; Wu et al., 2019; Zhang et al., 2019). It was reported that approximately 70% of medicinal herbs using tuberous roots were attacked by replant disease (Wu et al., 2016c). *R. glutinosa*, a member of the Scrophulariaceae family, is a traditional and famous Chinese medicinal herb with various pharmacological effects. However, 2-year consecutive monoculture of this plant on the same land led to a substantial increase in root rot disease and a serious reduction in tuberous root yield in the field trial conducted at our long-term orientation station. The consecutively- monocultured plants had unexpanded tuberous roots and large numbers of adventitious fibrous roots, which are of no commercial value (Wu et al., 2015, 2018b). In addition, consecutive monoculture of this plant resulted in a significant increase in the abundance of several soil-borne fungal pathogens (i.e., *Aspergillus flavus* and *Fusarium oxysporum*), which were frequently isolated from the consecutively monocultured soil and diseased plants and are pathogenic to *R. glutinosa* seedlings (Wu et al., 2015, 2016b, 2018a). Fields used for *R. glutinosa* production need to be planted with other crops for at least 15 years before they can be replanted again (Yang et al., 2011). Each year, replant disease causes a dramatic decrease in the area of the geo-authentic production zone (34° 48′ N to 35° 30′ N, 112° 02′ E to 113° 38′ E) (Zhang et al., 2019), an optimal production area with the most suitable soil and climate conditions for *R. glutinosa* cultivation. The quality of *R. glutinosa* tuberous roots cannot be assured when grown outside the geo-authentic production zone. Therefore, it is urgent to gain insight into the mechanisms underlying replant disease.

The plant microbiome is a key determinant of plant growth, development and health. The interactions between plants and soil microorganisms play key roles in maintaining soil quality and ecosystem sustainability (Kwak et al., 2018; Wang and Li, 2019). Plant roots influence soil microbial community assembly and alter the relative abundance of beneficial, harmful and neutral microorganisms, which in turn exert positive or negative effects on plant growth and resistance (Bakker et al., 2018; Stringlis et al., 2018; Zhalnina et al., 2018; Rolfe et al., 2019). Moreover, previous studies demonstrated that previous plants could affect the immunity and resistance of subsequent plant populations growing in the same soil through soil-borne microbial legacy (Bakker et al., 2018; Yuan et al., 2018; Kong et al., 2019). Therefore, recent research in the field of replant disease has increasingly focused on soil microbiome composition and function. Liu et al. (2019) indicated that consecutive soybean monoculture significantly decreased the fungal community diversity but increased the abundances of plant pathogens. Gao et al. (2019) found that consecutive sweet potato monoculture reduced the abundance of beneficial fungi such as *Chaetomium* but increased harmful fungi such as *Verticillium*, *Fusarium*, and *Colletotrichum*. Our previous study using barcoded pyrosequencing of 16S rDNA gene amplicons demonstrated that consecutive *R. glutinosa* monoculture modulated the rhizosphere microbiome with a reduction in the abundances of specific beneficial microorganisms and an increase in the harmful microorganisms (Wu et al., 2018b). Similar examples were also found for numerous medicinal plants including *Panax quinquefolius*, *P. notoginseng*, *P. heterophylla* and *P. ginseng* under a monoculture regime (Wu et al., 2016a; Zhao et al., 2017; Dong et al., 2018b; Jiang et al., 2019). Besides, a growing body of research has indicated that replant disease can be attributed to changes in the soil microbiome induced by phenolic allelochemicals, rather than their direct autotoxicity (Li et al., 2014; Wu et al., 2016a; Chen et al., 2017, 2018). Li et al. (2014) found that peanut root exudates could selectively stimulate or inhibit different microbial taxa, and the modifications in the soil microbiome mediated by phenolic acids led to the poor performance of the peanut plants. Our previous study found that the abundance of phenolic acids in *R. glutinosa* root exudates increased with the growth time of seedlings under sterile conditions but did not increase with the increasing years of monoculture under natural field conditions, suggesting that soil microbes might be involved in the degradation, utilization and conversion of root exudates (Wu et al., 2015).

The co-evolution between plants and associated microbial communities is common but complex in natural ecosystems (Zhang et al., 2017; Chagas et al., 2018). During coevolution with their host plant, microorganisms have evolved numerous strategies to talk with the hosts, intraspecific populations and other organisms for growth and survival (Badri et al., 2009; Venturi and Keel, 2016; Kan et al., 2017). Quorum sensing (QS) is a widespread phenomenon by which bacteria use intercellular communication to coordinate their behavior in response to environmental changes. Likewise, plant-associated bacteria utilize QS systems to sense the ecological niche, adapt to environmental stress, distribute their population under the existing conditions, and thereby influence the growth and health of host plants (Loh et al., 2002; Khan et al., 2019). N-acyl homoserine lactone (AHL)-mediated QS plays important roles in root-microbe interactions and the motility and colonization of rhizobacteria (Loh et al., 2002; von Bodman et al., 2003; Zhang et al., 2017). Plant root exudates not only provide nutrients and energy sources for root-associated microorganisms, but also select, attract or repel specific QS rhizobacteria (Bauer and Mathesius, 2004; Venturi and Keel, 2016; Chagas et al., 2018). Neal et al. (2012) found that 2,4-dihydroxy-7-methoxy-2H-1,4-benzoxazin-3(4H)-one (DIMBOA) released by maize roots could induce positive chemotaxis by *Pseudomonas putida* and attract these bacteria to the rhizosphere. Rosmarinic acid, a plant-derived compound that acts as a QS regulator agonist, activated the quorum sensing responses in *Pseudomonas aeruginosa* (Corral-Lugo et al., 2016). More interestingly, Schaefer et al. (2008) demonstrated that photosynthetic bacterium *Rhodopseudomonas palustris* had the ability to produce a novel QS signaling molecule, *p*-coumaroyl-homoserine lactone, by using a plant-derived aromatic acid, *p*-coumarate. In contrast, quorum quenching (QQ) refers to the process of interference in microbial QS (Dong et al., 2001; Uroz et al., 2009; Grandclement et al., 2016). Plant roots can also produce QS signal mimics or QS-interfering molecules to interfere with microbial QS systems (Teplitski et al., 2000; Zhang et al., 2017). Methyl gallate, a phenolic compound, was reported to inhibit both AHL synthesis and activity in *Chromobacterium violaceum* and to suppress biofilm formation and other QS-associated virulence factors in *P. aeruginosa*. In addition, microorganisms can utilize QQ strategies including the enzymatic degradation of QS molecules to prevent QS signaling and QS-regulated functions in plant-associated microorganisms.

Even though the rhizosphere microbiome plays crucial roles in maintaining soil health, the relationships between QS bacterial populations and replant disease are still unclear. A previous study on *P. heterophylla* replant disease indicated that the number of QS bacteria, all identified as *Serratia marcescen*s that can rapidly cause wilt disease, significantly increased with the increasing years of monoculture. Moreover, it was found that *P. heterophylla* root exudates and root tuber extracts could significantly promote the growth of *S. marcescens* (Zhang et al., 2016). Our previous studies have demonstrated that consecutive *R. glutinous* monoculture led to soil microbiome dysbiosis, and phenolic acids in root exudates could significantly promote the mycelial growth and toxin production of pathogenic *F. oxysporum* (Wu et al., 2015, 2018a,b). However, little is known about the shifts in QS bacterial populations in the *R. glutinous* rhizosphere under consecutive monoculture, as well as the effects of phenolic acids in root exudates on the growth of specific QS bacteria. We hypothesized that consecutive *R. glutinosa* monoculture could restructure the short-chain AHL-mediated QS bacterial populations in the rhizosphere through the modulation of root exudates, with an increase in the abundance of harmful QS bacteria but a reduction in beneficial QS bacteria.

## Materials and Methods

### Field Experiment and Soil Sampling

The field experiment was conducted in Jiaozuo city, Henan Province (34°56′N, 112°58′E), the geo-authentic production zone. The mean annual precipitation in this region is 552 mm, and the mean annual temperature is 14.3°C. *R. glutinosa* cultivar “Wen 85-5” was used as the experimental material in this study. To ensure uniform soil and climate conditions among different treatments, a single field previously cultivated with wheat was divided into two parts for two cropping patterns: the newly planted (NP) part and the 2-year consecutively monocultured (CM) part. The soil pH of the tested field was 7.43, and the soil organic matter content was 12.52 g⋅kg^–1^. The contents of available nitrogen, phosphorus and potassium were 23.41, 51.36, and 223.89 mg⋅kg^–1^, respectively. The total nitrogen, phosphorus and potassium were 0.51, 1.46, and 6.98 g⋅kg^–1^, respectively. In brief, *R. glutinosa* in the NP plots was planted on April 15 in 2016 and harvested on October 30 in 2016. The CM plots were established in 2015; the plots were consecutively monocultured for 2 years (Wu et al., 2018b). Each treatment had three experimental repetitions (12 m^2^). All study plots had the same fertilization and water management during the whole experimental period.

The rhizosphere soil samples were collected from 5 random locations within each plot on July 15 in 2016. *R. glutinosa* tuberous roots were carefully excavated with a shovel, shaken to remove loosely attached soil, and then tightly attached soil on the tuberous roots was collected as rhizosphere soils. For bacterial isolation, the soil samples from three replicates of each treatment were combined into a composite sample. All soil samples were passed through a 2-mm sieve to remove plant residues, macrofauna and stones and then used immediately for bacterial isolation and soil total DNA extraction.

### The Isolation and Identification of QS Bacteria From Rhizosphere Soil

The biosensor strain *Chromobacterium violaceum* CV026 (CV026), a mini-Tn5 mutant of *C. violaceum* ATCC31532 with kanamycin resistance (Sakr et al., 2013), was used to identify short-chain AHL-mediated QS bacteria (C4-C8-AHLs) (Remuzgo-Martínez et al., 2015). This strain cannot synthesize QS signal molecules but can sensitively respond to exogenous signal molecules (i.e., N-hexanoyl-l-homoserine lactone, C6-HSL) and produce purple violacein pigment (Hossain et al., 2017). The rhizosphere soils collected from healthy NP plants (denoted as NP soil) and the diseased CM plants with root rot disease symptoms (denoted as diseased soil, BT soil) were used to isolate short-chain AHL-mediated QS bacteria. Briefly, a serial dilution of soil suspension (10^–1^, 10^–2^, and 10^–3^) was perpared by using freshly collected soil. The soil suspension was spread on a plate containing Luria-Bertani (LB) medium and incubated at 37°C for 12 h. Then, 200 recognizable single colonies were randomly selected from an appropriate dilution (10^–3^ dilution) for each treatment because there were 200∼250 single colonies in this dilution on the LB agar plates. Each single colony was streaked and co-cultured with the biosensor CV026 on the LB plates at 30°C. The inoculation of a known non-QS bacterium and C6-HSL standard solution was used as a negative and positive control, respectively. The production of purple pigmentation by CV026 indicated that the co-cultured bacteria could produce QS signal molecules; these were identified as QS bacteria. The identified QS bacteria were stored at −80°C and were cultured overnight in LB medium for further use. The DNA of QS bacteria was extracted and then used for 16s rDNA amplification with the primer pair 357F (5′-CTCCTAGGGAGGCAGCAG-3′) and 1492r (5′-GGTTACCTTGTTACGACTT-3′) for Sanger sequencing. The sequence data have been submitted to GenBank (accession number MT355703∼MT355756).

### Quantitative PCR (qPCR) of *Pseudomonas* sp. in Rhizosphere Soil

The abundance of *Pseudomonas* in NP and BT soils was determined via qPCR with the specific primer pairs Ps-for (5′-GGTCTGAGAGGATGATCAGT-3′) and Ps-rev (5′-TTAGCTCCACCTCGC GGC-3′) (Tan and Ji, 2010). The PCR reaction mixture and amplification conditions were set as described by Wu et al. (2015). Each treatment had three biological replicates.

### Antagonistic Activity Assessment and Pathogenicity Test of QS Bacteria

The isolated QS bacteria were cultured overnight in LB medium at 37°C. The bacterial suspension was inoculated at the periphery of potato-dextrose-agar (PDA) plates and incubated for 2 days at 37°C. Then, the mycelium of *A. flavus* or *F. oxysporum*, two fungal causal agents of *R. glutinosa* root rot diseases (Wu et al., 2016b), was transferred to the center of the PDA plates and incubated again for several days to observe antagonistic activities of QS bacteria.

Simultaneously, QS bacteria were inoculated on Murashige-Skoog (MS) medium that was planted with tissue culture seedlings of *R. glutinosa* to assess the pathogenicity of QS bacteria. Firstly, seedlings shoots without roots were transferred to MS medium supplemented with 0.2 mg/L 6-benzyladenine, 0.2 mg/L indole-3- butyric acid and grown for 40 days. Then, the tissue culture seedlings inoculated with QS bacteria were cultured in a growth chamber at 25°C with a photoperiod of 16:8 h light/dark to observe the symptoms. Each treatment had three biological replicates.

### The Effects of Phenolic Acid Mixture on the Growth of QS Bacteria

Based on the detection of the composition and total abundance of phenolic acids in the *R. glutinosa* rhizosphere (Wu et al., 2015), a phenolic acid mixture at the same ratio (molar ratio, protocatechuic acid : phthalic acid : *p*-hydroxybenzoic acid : vanillic acid : syringic acid : vanillin : ferulic acid : benzoic acid = 10 : 10 : 36 : 100 : 12 : 12 : 30 : 30) was applied to assess the effects of phenolic acids on the chemotactic response and growth of QS bacteria. The chemotaxis of QS bacteria was performed using the drop assay described by Li et al. (2012) with some modification. In detail, 500 μL of QS bacterial suspension was added into 20 mL of minimal medium (Rani et al., 1996) and poured into a petri plate. Subsequently, 0.1 g of phenolic acid powder mixture at the above-mentioned ratio was placed in the center of a petri plate and chemotactic response was observed via migrating rings after 24 h of incubation at 37°C. The growth response of QS bacteria to phenolic acids was assessed through the optic density (OD) value assay. Briefly, the stock solution of phenolic acids was filtered through 0.22 μm filters and then added to an 8-fold dilution of LB broth (1/8 LB) medium to prepare a series of solutions with different final concentrations (0, 30, 60, 120, 240, 480 μmol⋅L^–1^). Each treatment had three replicates. Thirty μL of QS bacteria was inoculated into 1/8 LB medium. Afer incubation at 200 rpm and 37°C for 8 h, the OD value at 600 nm (OD_600_) was detected using a plate reader (Thermo Scientific Multiskan MK3, Shanghai, China).

### The Isolation of QQ Bacteria That Could Disrupt the *Pseudomonas brassicacearum* SZ50 QS System

The QS bacteria belonging to the *Pseudomonas* genus were widespread in NP soil in this study and showed a declining trend under consecutive *R. glutinosa* monoculture. Therefore, *P. brassicacearum*, a *Pseudomonas* baterium showing strong antagonistic activity against the fungal pathogen *A. flavus*, was selected as a target to isolate the corresponding QQ bacteria. In addition to NP and BT soils, another soil (denoted as consecutively cropped soil, CC soil) collected from 2-year CM plants, which had high numbers of adventitious fibrous roots but no root rot disease symptoms, was used for QQ bacterial isolation.

The biosensor strain CV026 was used to assess QQ activity against *P. brassicacearum* SZ50 and to isolate QQ bacteria from NP, CC and BT soils. Firstly, the *P. brassicacearum* SZ50 strain was cultured in LB medium for 16 h, and the QS signal molecules (AHL) were extracted using acidified ethyl acetate (supplemented with 1% acetic acid) according to the method of Anbazhagan et al. (2012) with modifications. AHL extracts were dissolved in HPLC-grade methanol and qualitatively detected by the well-diffusion assay using biosensor CV026 (Anbazhagan et al., 2012). Subsequently, the resulting AHL extracts were used for QQ bacterial isolation. In detail, the isolated strains were grown in sterile 96-well plates (plate No. 1) containing 300 μl of 1/2 TY broth (0.5% tryptone, 0.3% yeast extract, 15 mM KH_2_PO_4_ and 6 mM CaCl_2_) at 200 rpm and 37°C for 16 h. Then, 50 μL culture broths of the isolated strains were transferred into each well of sterile 96-well plates (plate No. 2) containing 50 μl of 1/2 TY broth supplemented with 1 μL of *P. brassicacearum* AHL extracts and incubated at 300 rpm and 30°C for 24 h. Wells containing 50 μL culture broth of a known QQ bacterium, 50 μl of 1/2 TY broth and 1 μl of C6-HSL were used as a positive control. Wells containing only 100 μl of 1/2 TY broth supplemented with 1 μl of AHL extracts or C6-HSL were both used as a negative control. After ultraviolet (UV) sterilization for 30 min, 50 μl of the sterilized culture broths were transferred into each well in new 96-well plates (plate No. 3) containing 100 μl of 1/2 TY broth, 50 μL of overnight-cultured CV026 and 0.18 μL kanamycin (50 μg⋅mL^–1^) and incubated at 300 rpm and 30°C for 24 h to observe the QQ activity of the isolated strains. No purple pigmentation produced by CV026 in plates No. 3 indicated that the corresponding bacteria in plate No. 1 could degrade *P. brassicacearum* AHL signal molecules and could be identified as QQ bacteria (Supplementary Figure S1). The QQ candidates were checked again through the above-mentioned method. Similarly, the molecular identification of QQ bacteria was performed by using 357F and 1492r as mentioned above. The sequence data have been submitted to GenBank (accession numbers MT355757∼MT355785).

### The Functional Characterization of Specific QQ Bacteria

The effects of phenolic acid mixture on the growth of specific QQ bacteria and their pathogenicity test were carried out using the same method as described above for QS bacteria.

### Statistical Analyses

Statistical analysis of data was performed by one-way analysis of variance (ANOVA) followed by the least significant difference (LSD) test (*P* < 0.05, *n* = 3) via Data Processing System (DPS) software (version 7.05, Zhejiang University, Hangzhou, China). Phylogenetic tree construction was performed with Molecular Evolutionary Genetics Analysis (MEGA) version 4.1.

## Results

### Changes in the Species Composition and Population Dynamics of Short-Chain AHL-Mediated QS Bacteria in the Rhizosphere Under Consecutive Monoculture

**FIGURE 1:**
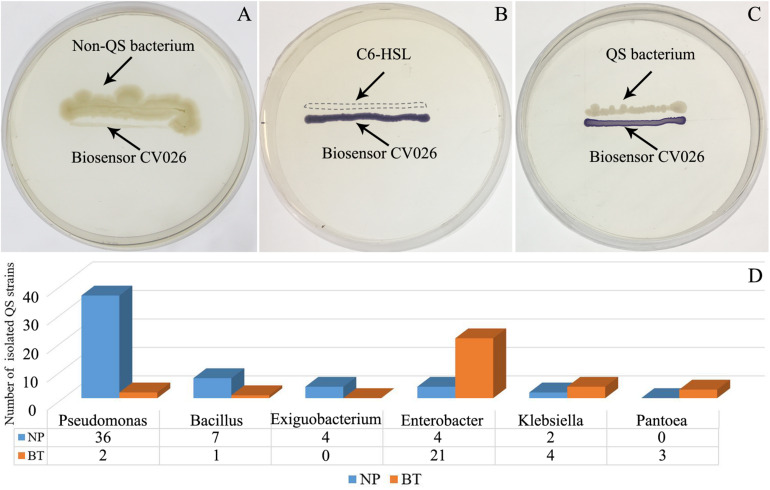
Screening of short-chain AHL-mediated QS bacteria by biosensor strain CV026 **(A–C)** and the dynamic changes of QS bacteria in the *R. glutinosa* rhizosphere under consecutive monoculture **(D)**. **(A)** The biosensor strain CV026 did not produce the purple pigmentation when co-cultured with a known non-QS bacterium. **(B)** CV026 produced the purple pigmentation when inoculated with C6-HSL standard, as indicated by a dashed box. **(C)** The CV026 colony developed a purple color when co-cultured with a QS bacterium randomly isolated from the *R. glutinosa* rhizosphere.

**FIGURE 2:**
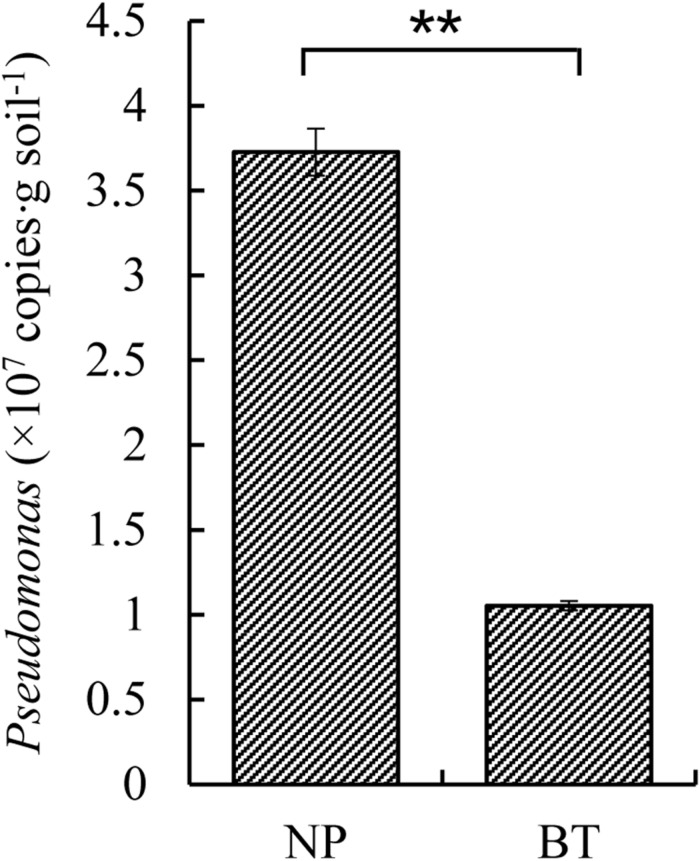
Quantitative PCR of *Pseudomonas* sp. in the newly planted (NP) soil and the diseased (BT) soil of *R. glutinosa*. ** indicates a statistically significant difference (*P* < 0.01, LSD test). Data are presented as means ± standard errors.

Compared with NP, CM plants had serious rot root disease and unexpanded tuberous roots. Moreover, 62% of consecutively- monocultured plants withered and died during sampling. The soil samples collected from the NP and diseased CM plants were used for QS bacterial isolation. Co-cultured with a non-QS bacterium, the biosensor strain CV026 did not produce purple pigmentation (Figure 1A). However, the positive control inoculated with the C6-HSL standard, the CV026 colony developed a deep purple color (Figure 1B). Based on this principle, the short-chain AHL-mediated QS bacteria were randomly isolated by biosensor CV026 from the newly planted (NP) soil and the diseased (BT) soil (Figure 1C). The results showed that 65 out of 200 strains (32.5%) randomly selected from the newly planted (NP) soil of *R. glutinosa* were detected as QS bacteria, mainly consisting of *Pseudomonas* spp. (55.4%). By contrast, 34 out of 200 (17%) strains from the diseased (BT) soil were detected as QS bacteria, mainly consisting of Enterobacteriaceae (73.5%), including *Enterobacter* spp. (61.8%) and *Klebsiella* spp. (11.8%). The phylogenetic trees of 16S rDNA genes of QS bacteria isolated from NP and BT soils are shown in Supplementary Figures S2, S3, respectively. Moreover, the number of QS bacteria belonging to *Pseudomonas*, *Bacillus* and *Exiguobacterium* was markedly higher in the NP soil than in the BT soil. However, the number of QS bacteria belonging to *Enterobacter*, *Klebsiella* and *Pantoea* increased under consecutive *R. glutinosa* monoculture (Figure 1D). Quantitative PCR confirmed that the abundance of the *Pseudomonas* genus was significantly higher in the NP soil (3.7 × 10^7^ copies ⋅ g soil^–1^) than in the BT soil (1.1 × 10^7^ copies ⋅ g soil^–1^) (Figure 2).

### Antagonistic Activity Assessment and Pathogenicity Test of QS Bacteria

**FIGURE 3:**
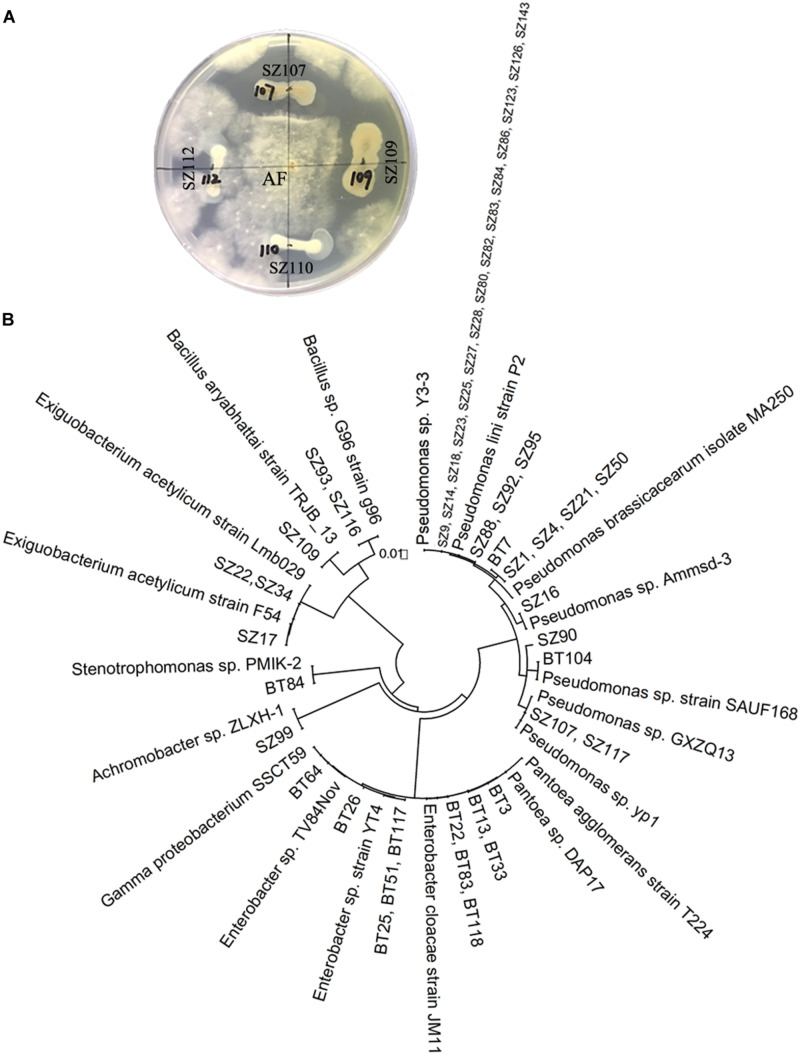
Antagonistic activity assessment of QS bacteria **(A)** and phylogenetic analysis of QS antagonists **(B)**. **(A)** Strains SZ107 and SZ109, rather than SZ110 and SZ112, had antagonistic activities against *A. flavus* (AF). The prefixes “SZ” and “BT” indicate the QS bacteria isolated from the newly planted (NP) soil and the diseased (BT) soil, respectively.

The antagonistic activity assessment showed that 33 out of 65 strains of QS bacteria (50.8%) in the NP soil had antagonistic activities against *A. flavus* or *F. oxysporum*, two fungal causal agents of *R. glutinosa* root rot diseases. In particular, most of the QS bacteria belonging to *Pseudomonas* (72.2%) had inhibitory effects on the growth of *A. flavus*. Further, several strains such as *Pseudomonas* spp. SZ88, SZ92, SZ95, SZ16, SZ90, and *Bacillus* sp. SZ93 possessed antagonistic activities against both fungal pathogens (Supplementary Table S1 and Figure 3). By contrast, 14 out of 34 strains of QS bacteria (41.2%) in the BT soil showed antagonistic activity against one of the two fungal pathogens. Except for BT7 with antagonistic activity against *A. flavus*, the others only showed antagonistic activity against *F. oxysporum* (Supplementary Table S1 and Figure 3). Morover, the dominant QS bacteria in the BT soil, such as *Pantoea* sp. BT3, *Enterobacter* sp. BT22, *Enterobacter* sp. BT23, and *Enterobacter* sp. BT56, were highly pathogenic to the tissue culture seedlings of *R. glutinosa* (Supplementary Figure S4).

### Different Responses of Beneficial and Harmful QS Bacteria to *R. glutinosa* Root Exudates

Based on the phylogenetic trees of isolated QS bacteria (Supplementary Figures S2, S3), most of the dominant taxa were selected to test their chemotactic responses toward phenolic acids identified in root exudates of *R. glutinosa* through the drop assay. The results showed that most of the *Enterobacter* spp. and *Klebsiella* spp., frequently isolated from the BT soil, exhibited distinct chemotaxis toward the phenolic acid mixture. However, several QS strains including *Pseudomonas* sp. SZ90, *Bacillus* sp. 31, *Bacillus* sp.77, *Exiguobacterium* sp. SZ17 and *Achromobacter* sp. SZ99 showed no appreciable chemotaxis (Supplementary Figure S5).

**FIGURE 4:**
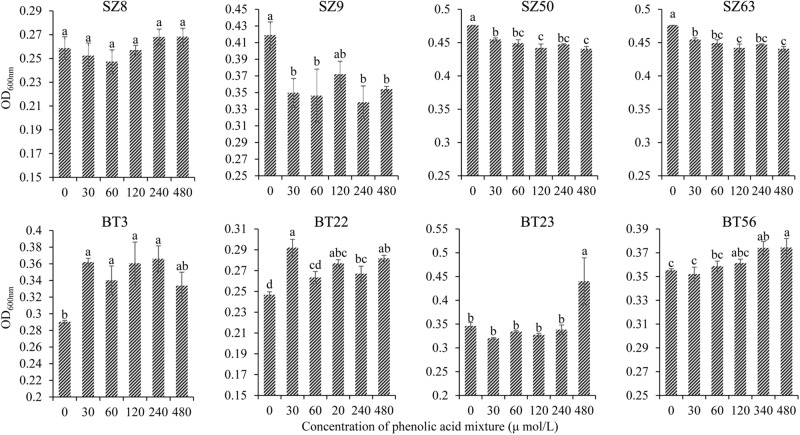
Effects of phenolic acid mixture on the growth of specific QS bacteria isolated from the newly planted (NP) soil and the diseased (BT) soil of *R. glutinosa*. The prefixes “SZ” and “BT” indicate the QS bacteria isolated from the NP soil and the BT soil, respectively. Strains S8, S9, SZ50, and SZ63 belong to the *Pseudomonas* genus. Strain BT3 belongs to the *Pantoea* genus, strains BT22 and BT23 belong to the *Enterobacter* genus and strain BT56 belongs to the *Klebsiella* genus. Data are presented using means ± standard errors. Different letters indicate different levels of significance (*P* < 0.05, LSD, *n* = 3).

Furthermore, the different responses of specific beneficial and harmful QS bacteria to the phenolic acid mixture were assessed through the OD value assay. The results showed that the phenolic acid mixture at the same ratio as detected in *R. glutinosa* rhizosphere soil had concentration-dependent inhibitory effects on the growth of beneficial QS bacteria including *Pseudomonas* spp. S9, SZ50, and SZ63, but not SZ8, that were dominant in the NP soil. However, the phenolic acid mixture significantly promoted the growth of harmful QS bacteria that were dominant in the BT soil including *Pantoea* sp. BT3, *Enterobacter* spp. BT22 and BT23, and *Klebsiella* sp. BT56, and the promotion effect increased as the concentration increased (Figure 4).

### Dynamic Change in Bacteria With Quorum Quenching Activity Against Beneficial QS Bacteria Under Consecutive Monoculture

**FIGURE 5:**
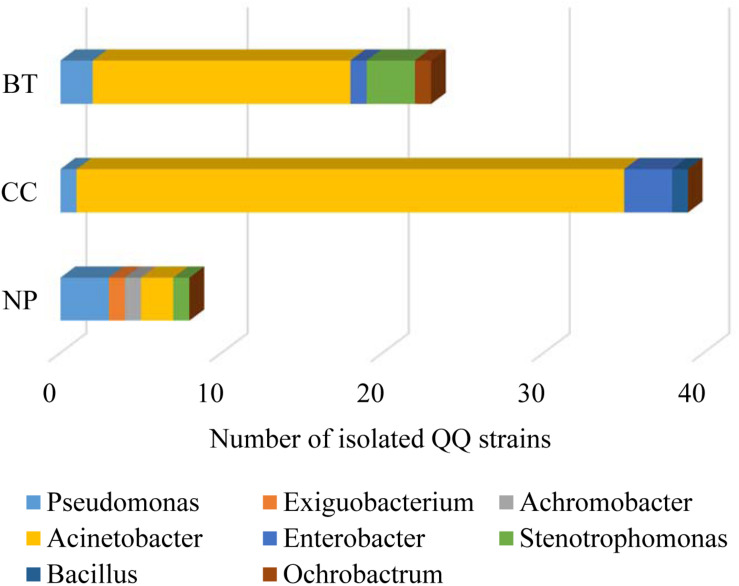
The species composition and population dynamics of rhizosphere bacteria with QQ activities against *Pseudomonas* SZ50 under consecutive *R. glutinosa* monoculture. NP, CC, and BT represent the newly planted soil, the 2-year consecutively cropped soil and the diseased soil, respectively.

The well-diffusion assay using biosensor CV026 demonstrated that QS signal molecules (AHLs) were successfully extracted from the culture broth of *P. brassicacearum* SZ50 strain (Supplementary Figure S6). Screening of QQ bacteria with enzymatic degradation activity against SZ50 AHLs showed that 8 out of 200 strains (4.0%) randomly selected from the NP soil were detected as QQ bacteria, consisting of *Pseudomonas* (37.5%), *Exiguobacterium* (12.5%), *Achromobacter* (12.5%), *Acinetobacter* (25.0%), and *Stenotrophomonas* (12.5%). However, 39 out of 200 strains (19.5%) and 23 out of 200 strains (11.5%) were identified as QQ bacteria in the CC soil and BT soil, respectively. Moreover, QQ bacteria in the consecutively monocultured soils mainly consisted of *Acinetobacter* spp., accounting for 87.2% (34 strains) in CC soil and 69.6% (16 strains) in BT soil (Figure 5). Further, several QQ bacteria belonging to *Enterobacter* spp. were detected in the consecutively monocultured soils, including *Enterobacter* sp. YQC23, *Enterobacter* sp. YQC24, and *Enterobacter* sp. YQC119. The phylogenetic trees of the 16S rDNA genes of QQ bacteria isolated from NP, CC, and BT soils are shown in Supplementary Figure S7.

Furthermore, the effects of phenolic acids on the growth of specific QQ bacteria isolated from the consecutively monocultured soil were tested by OD value assay. The results showed that the phenolic acid mixture at the same ratio as that detected in *R. glutinosa* rhizosphere soil could significantly increase the growth of specific QQ bacteria belonging to *Acinetobacter* spp. (i.e., YQC11, YQC12, and YQB98) and *Enterobacter* spp. (i.e., YQC23, YQC24, and YQC119) (Supplementary Figure S8), which were isolated from the consecutively monocultured soils. Furthermore, a pathogenicity test showed that these specific QQ bacteria isolated from the CC and BT soils could cause severe wilt disease in the tissue culture seedlings of *R. glutinosa* (Supplementary Figure S9).

## Discussion

The collective genome of the rhizosphere microbial community is referred to as the second genome of the plant and plays crucial roles in plant growth and health (Berendsen et al., 2012). A growing body of research has demonstrated that replant disease is closely associated with changes in the rhizosphere microbiome (Franke-Whittle et al., 2015; Chen et al., 2017; Yu et al., 2019). Plant roots release variable but substantial amounts of compounds and can shape the rhizosphere microbial community structure (Hu et al., 2018; Stringlis et al., 2018; Voges et al., 2019). Soil microorganisms can actively respond to environmental changes, alter their physiological behavior including motility and chemotaxis, and even modulate the plant root exudation profile (Matilla et al., 2010; Gu et al., 2016; Zhalnina et al., 2018). Quorum sensing, a cell density-dependent type of intercellular communication, allows bacteria to monitor the surrounding environment, coordinate the behavior of populations, and mediate microbe-microbe and host-microbe interactions (Loh et al., 2002; Taga and Bassler, 2003; Chagas et al., 2018). AHL-mediated QS regulates diverse behavior involving both intra- and interspecies interactions in rhizosphere-inhabiting and plant pathogenic bacteria (von Bodman et al., 2003; Zaytseva et al., 2019).

In this study, the species composition and population dynamics of QS bacteria driven by consecutive monoculture of *R. glutinosa* were explored. The results showed that numerous bacteria in soil were detected as QS bacteria, accounting for 32.5% of all randomly selected strains in the NP soil and 17% in the diseased soil. This observation is consistent with the findings in previous studies that QS systems are widespread among the bacterial populations in the phytosphere (Veselova et al., 2003; Zhang et al., 2016). Molecular identification found that the QS bacteria isolated from the healthy NP soil mainly consisted of *Pseudomonas* spp. (accounting for 55.4%) and *Bacillus* spp. (accounting for 10.8%) (Figure 1D). Quantitative PCR confirmed the significant decline in *Pseudomonas* spp. in the *R. glutinosa* rhizosphere under consecutive monoculture (Figure 2). Moreover, more than half of the QS bacteria in the NP soil showed antagonistic activities against *A. flavus* or *F. oxysporum*, two fungal causal agents of *R. glutinosa* root rot diseases (Figure 3). Similar findings were reported in our previous study on the rhizosphere microbiome of *R. glutinosa* based on barcoded pyrosequencing (Wu et al., 2018b). Luo et al. (2019) indicated that *P. notoginseng* planting resulted in negative plant-soil feedback due to a decline in the beneficial rhizobacteria including the genera *Pseudomonas* and *Bacillus* in the rhizosphere. *Pseudomonas* spp. and *Bacillus* spp. have been widely proposed to play important roles in specific soil suppressiveness to plant pathogens (Mendes et al., 2011; Kyselková and Moënne-Loccoz, 2012; Shen et al., 2015; Zhou et al., 2019). By contrast, the QS bacteria isolated from the unhealthy BT soil mainly consisted of *Enterobacter* spp. (61.8%) and *Klebsiella* spp. (11.8%) (Figure 1D), and some of them were found to be pathogenic to *R. glutinosa* seedlings. Similarly, Lin et al. (2015) found that the abundance of *Enterobacter* spp. significantly increased in *P. heterophylla* rhizosphere soil with increasing years of monoculture. Köberl et al. (2017) indicated that *F. oxysporum*-infected banana plants harbored a higher abundance of Enterobacteriaceae known for their plant-degrading capacity. However, it should be noted that the culture-independent approaches in our previous study found that the relative abundance of Enterobacteriaceae was significantly higher in newly planted soil than in consecutively monocultured soil (Wu et al., 2018b), which might be due to the difference between culture-dependent and culture-independent assessments. Moreover, the CV026 reporter strain used in this study mainly detects short-chain AHLs (Remuzgo-Martínez et al., 2015). These results suggested that the imbalance between beneficial and harmful QS bacteria in the rhizosphere under consecutive monoculture might be an important factor for *R. glutinosa* replant disease. Further studies are needed to assess the effects of the synthetic multispecies community created using the isolated bacteria on the growth and development of tuberous roots of *R. glutinosa* in natural field sites.

Root exudates are the key determinant of rhizosphere microbiome assembly, acting as chemical attractants, repellants or antagonists of specific microorganisms in soil (el Zahar Haichar et al., 2014; Huang et al., 2014; Chagas et al., 2018; Stringlis et al., 2018). Hu et al. (2018) demonstrated that plant root exudates could modulate the rhizosphere microbiota and thereby affect the growth and defense of the next plant generation. Here, it was found that the phenolic acid mixture at the same ratio as that detected in *R. glutinosa* rhizosphere soil showed concentration-dependent inhibitory effects on the growth of several beneficial QS bacteria (i.e., *Pseudomonas* spp.) but promoted the growth of harmful QS bacteria (i.e., *Enterobacter* spp.) (Figure 4). This result is in line with previous findings that phenolic acids in *P. heterophylla* root exudates could selectively inhibit beneficial microorganisms (i.e., *Pseudomonas* spp.) and stimulate certain pathogenic bacteria and fungi (Wu et al., 2016c; Chen et al., 2017). Zhang et al. (2016) also demonstrated that both root exudates and tuberous root extracts of *P. heterophylla* could significantly promote the growth of *S. marcescens*, a QS bacterium that rapidly causes wilt disease of *P. heterophylla*. Numerous studies indicated that indirect allelopathy through modifications in soil microbiome induced by root exudates contributes to replant disease in agriculture and horticulture (Li et al., 2014; Chen et al., 2018). Besides, our findings indicated that the QS system of *Pseudomonas* sp. was disrupted by specific QQ bacteria in the soil via the enzymatic destruction of signal molecules. Moreover, the number of bacteria with QQ activity against *P. brassicacearum* SZ50 was considerably higher in the CC soil and BT soil than in the NP soil, and the number of QQ bacteria belonging to *Acinetobacter* spp. greatly increased in the rhizospehre soil under consecutive *R. glutinosa* monoculture (Figure 5). The AHL-degrading activity of *Acinetobacter* spp. has been widely documented in previous studies (Kang et al., 2004; Chan et al., 2011; Ochiai et al., 2013). The QS system is known to play a pivotal role in regulating diverse behavior and functions, such as rhizosphere colonization and competence and biocontrol activities (Loh et al., 2002; Venturi and Keel, 2016). Therefore, QS disruption might be another factor contributing to a decline in antagonistic *Pseudomonas* spp. in the *R. glutinosa* rhizosphere under consecutive monoculture.

In conclusion, consecutive monoculture of *R. glutinosa* resulted in negative soil-borne legacy effects including the build-up of potentially harmful short-chain AHL-mediated QS bacteria in the rhizosphere and the reduction of beneficial short-chain AHL-mediated QS bacteria, which was not only mediated by specific root exudates but also by QQ bacteria. The findings in this work indicated the importance of the rhizosphere microbiome in maintaining soil health and highlighted a link between short-chain AHL-mediated quorum sensing and *R. glutinosa* replant disease. However, further studies will be performed to investigate the roles of rhizosphere bacteria producing the long-chain AHLs in replant disease and the functions of QQ bacteria that target QS signaling of other strains. In addition, further work is needed to restore soil health and overcome the replant disease of *R. glutinosa* by quorum quenching.

## Data Availability Statement

All datasets generated for this study are included in the article/Supplementary Material.

## Author Contributions

LW and WL conceived the study. LW and QL wrote the manuscript. LW, YW, QL, JW, BY, JC, and HW performed the experiments. LW, BY, and QL performed the statistical analyses. JC, ZZ, and CL were involved in the field management and soil sampling. All authors discussed the results and commented on the manuscript.

## Conflict of Interest

The authors declare that the research was conducted in the absence of any commercial or financial relationships that could be construed as a potential conflict of interest.
