# Supplementary material for: Linking Short-Chain N-Acyl Homoserine Lactone-Mediated Quorum Sensing and Replant Disease: A Case Study of Rehmannia glutinosa
Source: Front Plant Sci. 2020 Jun 17;11:787. doi: 10.3389/fpls.2020.00787 (PMC7311668; doi:10.3389/fpls.2020.00787)
Supplement: Supplementary file 1 [file Data_Sheet_1.doc]

**Linking** Short-chain N-acyl Homoserine Lactone-mediated **Quorum Sensing and Replant Disease: A Case Study of *Rehmannia glutinosa***

***Qian Li^1,2†^, Yanhong Wu^1,2†^,*** ***Juanying Wang^1,2^, Bo Yang^1,2^, Jun Chen^1,2^, Hongmiao Wu^1,2^, Zhongyi Zhang^3^, Cuihong Lu^4^, Wenxiong Lin******^1^,******^5*^* and *Linkun Wu^1,2*^***

*^1^* *College of Life Sciences, Fujian Agriculture and Forestry University, Fuzhou, China.*

*^2^ Key Laboratory of Crop Ecology and Molecular Physiology, Fujian Agriculture and Forestry University, Fuzhou, China.*

*^3^ College of Crop Science, Fujian Agriculture and Forestry University, Fuzhou, China.*

*^4^ Wenxian Institute of Agricultural Sciences, Jiaozuo, China.*

*^5^* *Fujian Provincial Key Laboratory of Agroecological Processing and Safety Monitoring, Fujian Agriculture and Forestry University, Fuzhou, China.*

*Correspondence:

Linkun Wu; [wulinkun619@163.com](mailto:wulinkun619@163.com)

Wenxiong Lin; [lwx@fafu.edu.cn](mailto:lwx@fafu.edu.cn)

***^†^*** These authors have contributed equally to this work.

**SUPPLEMENTARY MATERIAL**

**Supplementary FIGURE S1 | Screening of QQ bacteria using the biosensor strain** ***Chromobacterium violaceum* CV026 and 96-well plates.** A: Wells containing 1/2 TY broth, a known QQ bacterium, C6-HSL and CV026 were used as a positive control. Biosensor CV026 did not produce the purple pigmentation because the known QQ bacterium could degrade the C6-HSL. B: Wells containing only 1/2 TY broth, C6-HSL and CV026 were used as a negative control. C: Wells containing only 1/2 TY broth, AHL extracts and CV026 were used as a negative control as well. D: Wells contained 1/2 TY broth, a randomly isolated strain, C6-HSL, and CV026. No purple pigmentation production indicated that the randomly isolated strains in corresponding wells were QQ strains, as indicated by the red box.

**Supplementary FIGURE S2 | The species composition of short-chain AHL-mediated QS bacteria isolated from the newly planted soil of *R. glutinosa* (A) and their phylogenetic analysis (B).** A: The number in the brackets represented the number of isolated QS strains belonging to the corresponding genera. B: The prefixes “SZ” indicate the QS bacteria isolated from the newly planted soil.

**Supplementary FIGURE S3 | The species composition of short-chain AHL-mediated QS bacteria isolated from the diseased soil of *R. glutinosa* (A) and their phylogenetic analysis (B).** A: The number in the brackets represented the number of isolated QS strains belonging to the corresponding genera. B: The prefixes “BT” indicate the QS bacteria isolated from the diseased soil.

**Supplementary FIGURE S4 | Assessment of the pathogenicity of specific QS bacteria isolated from the diseased soil.** CK: Equal amount of LB broth medium was used as a negative control (CK). The prefixes “BT” indicate the QS bacteria isolated from the diseased soil.

**Supplementary FIGURE S5 | Assessment of the chemotaxis of specific QS bacteria isolated from the *R. glutinosa* rhizosphere soil by drop assay.** The prefixes “SZ” and “BT” indicate the QS bacteria isolated from the NP soil and the BT soil, respectively. The name before the petri dishes indicated the taxonomic classification of QS bacteria in the same row. More details about the molecular identification of QS bacteria were shown in Supplementary Figures S2 and S3.

**Supplementary FIGURE S6 | Detection of QS signal molecules (AHLs) extracted from the culture broth of *Pseudomonas brassicacearum* SZ50 by biosensor CV026 through well-diffusion assay.** The left petri dish containing 6 μL of 10 μmol·mL^-1^ N-hexanoyl-l-homoserine lactone (C6-HSL) in the well was used as a positive control.

**Supplementary FIGURE S7 | The phylogenetic trees of 16S rDNA genes of QQ bacteria isolated from the newly planted soil (NP), the two-year consecutively cropped soil (CC) and the diseased soil (BT).** The prefixes “YQZ”, “YQC” and “YQB” indicate the QS bacteria isolated from the NP soil, the CC soil and the BT soil, respectively. B: YQC group includes strains YQC15, YQC18, YQC33, YQC34, YQC41, YQC42, YQC43, YQC44, YQC45, YQC47, YQC48, YQC49, YQC50, YQC60, YQC61, YQC68, YQC74, YQC75, YQC79, YQC84, YQC117 and YQC120.

**Supplementary FIGURE S8 | Effects of the phenolic acid mixture on the growth of specific QQ bacteria isolated from the two-year consecutively cropped soil (CC) and the diseased soil (BT) of *R. glutinosa*.** The prefixes “YQC” and “YQB” indicate the QQ bacteria isolated from the CC soil and the BT soil, respectively. Data are presented as means ± standard errors.

**Supplementary FIGURE S9 | Assessment of the pathogenicity of specific QQ bacteria isolated from the two-year consecutively cropped soil (CC) and the diseased soil (BT).** CK: Equal amount of LB broth medium was used as a negative control (CK). The prefixes “YQC” and “YQB” indicate the QQ bacteria isolated from the CC soil and the BT soil, respectively.

**Supplementary TABLE S1 | Antagonistic activity assessment of QS bacteria isolated from the newly planted soil and the diseased soil.**


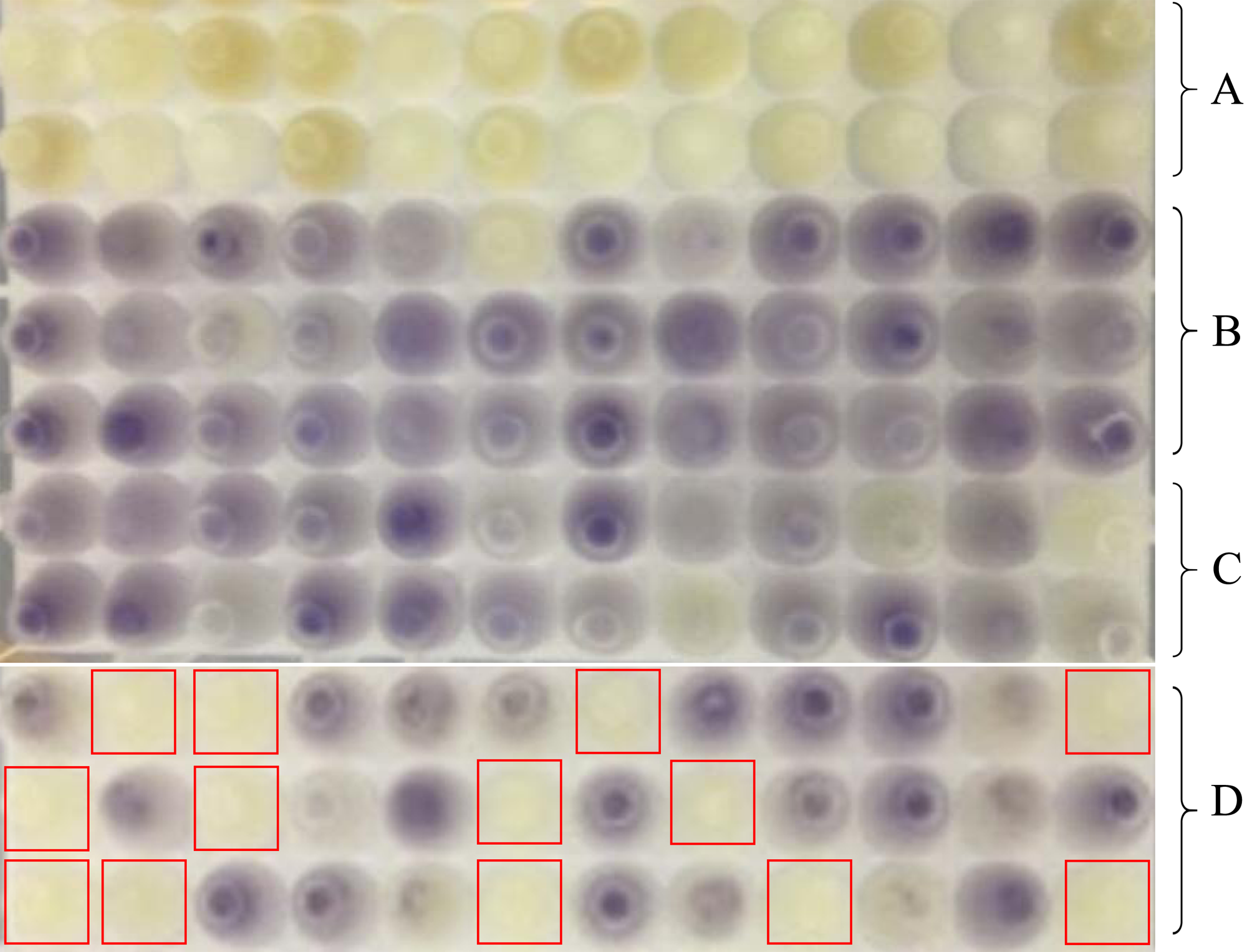


**Supplementary FIGURE S1 | Screening of QQ bacteria using the biosensor strain *Chromobacterium violaceum* CV026 and 96-well plates.** A: Wells containing 1/2 TY broth, a known QQ bacterium, C6-HSL and CV026 were used as a positive control. Biosensor CV026 did not produce the purple pigmentation because the known QQ bacterium could degrade the C6-HSL. B: Wells containing only 1/2 TY broth, C6-HSL and CV026 were used as a negative control. C: Wells containing only 1/2 TY broth, AHL extracts and CV026 were used as a negative control as well. D: Wells contained 1/2 TY broth, a randomly isolated strain, C6-HSL, and CV026. No purple pigmentation production indicated that the randomly isolated strains in corresponding wells were QQ strains, as indicated by the red box.


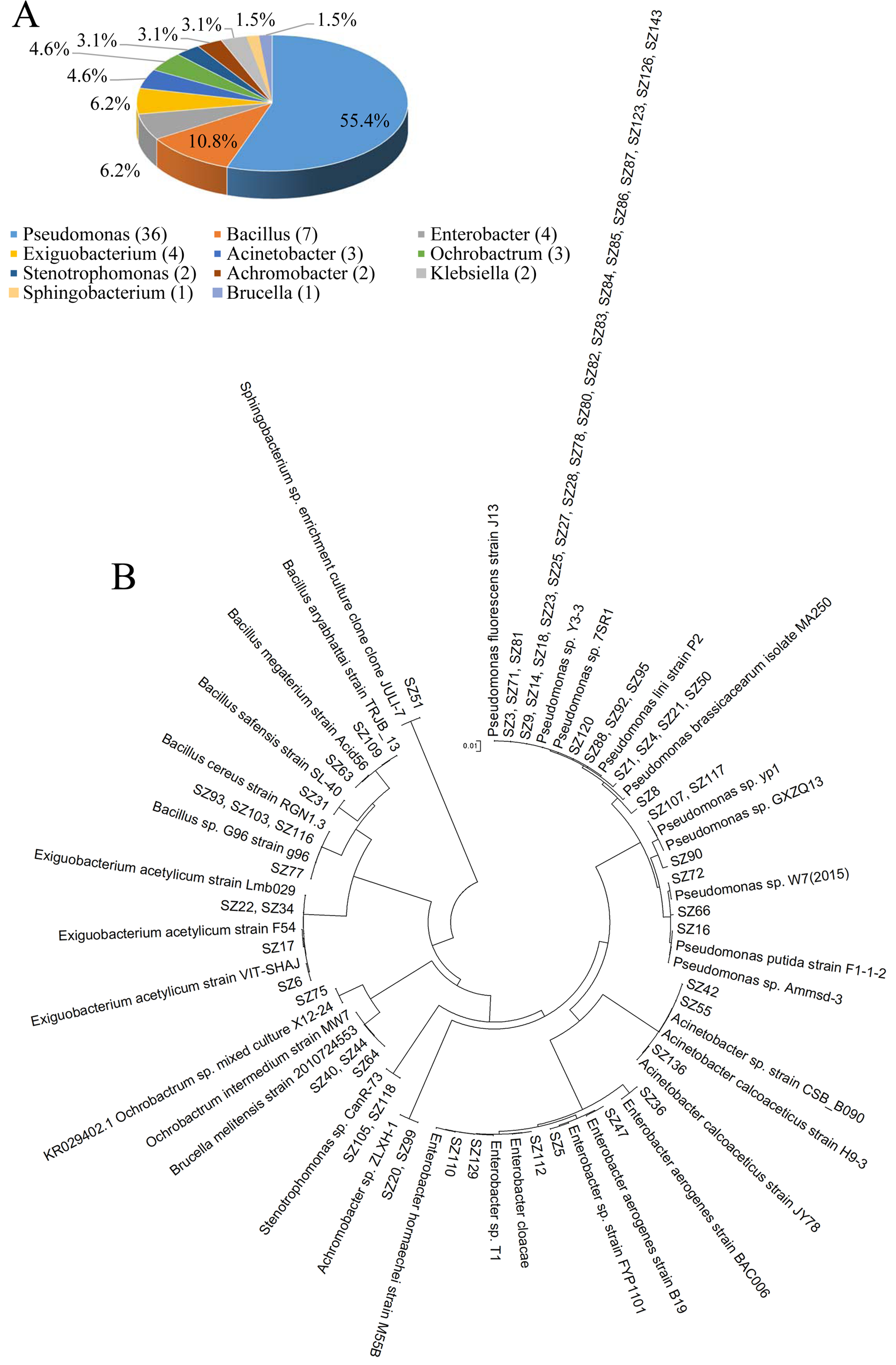


**Supplementary FIGURE S2 | The species composition of short-chain AHL-mediated QS bacteria isolated from the newly planted soil of *R. glutinosa* (A) and their phylogenetic analysis (B).** A: The number in the brackets represented the number of isolated QS strains belonging to the corresponding genera. B: The prefixes “SZ” indicate the QS bacteria isolated from the newly planted soil.


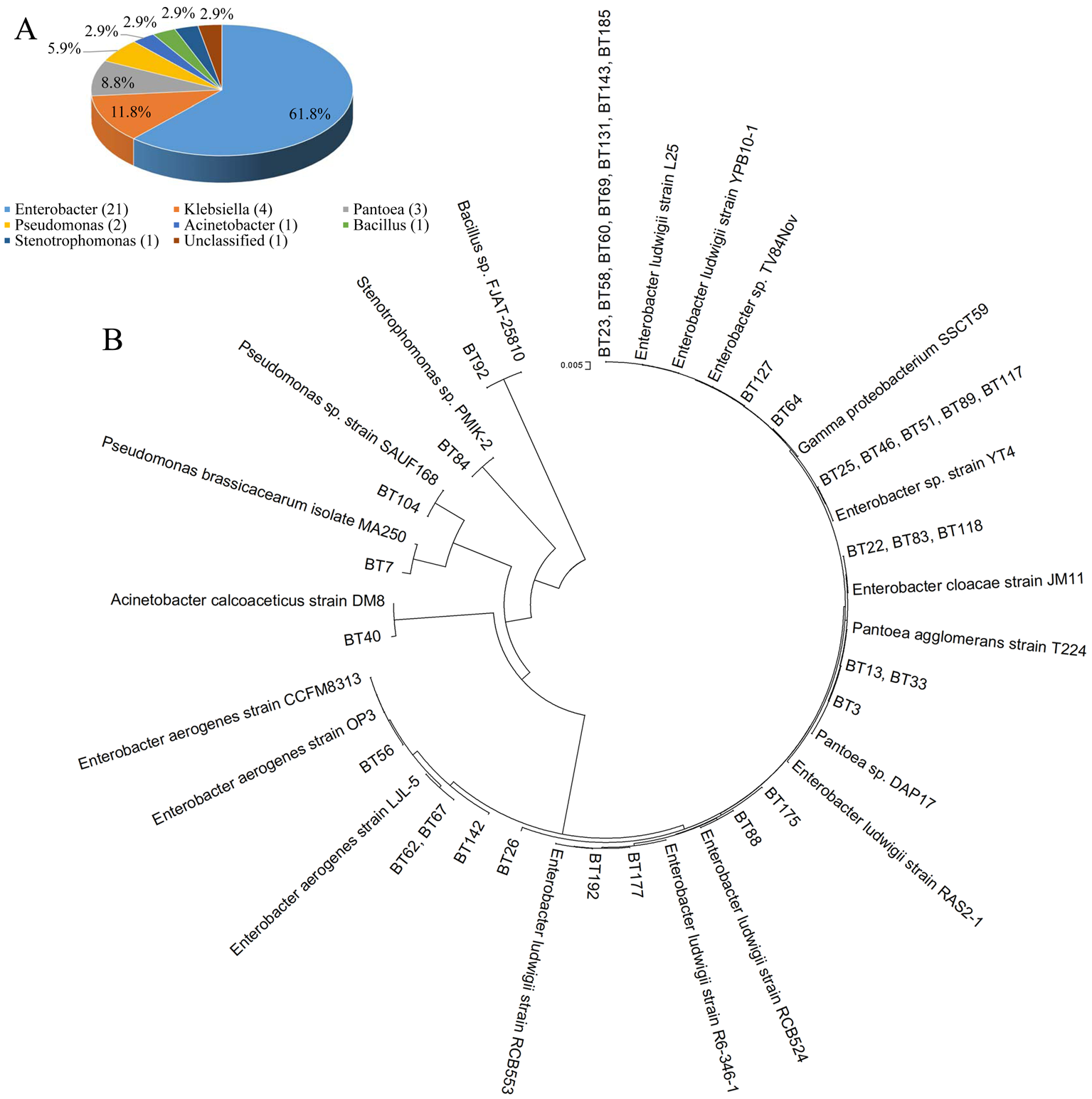


**Supplementary FIGURE S3 | The species composition of short-chain AHL-mediated QS bacteria isolated from the diseased soil of *R. glutinosa* (A) and their phylogenetic analysis (B).** A: The number in the brackets represented the number of isolated QS strains belonging to the corresponding genera. B: The prefixes “BT” indicate the QS bacteria isolated from the diseased soil.


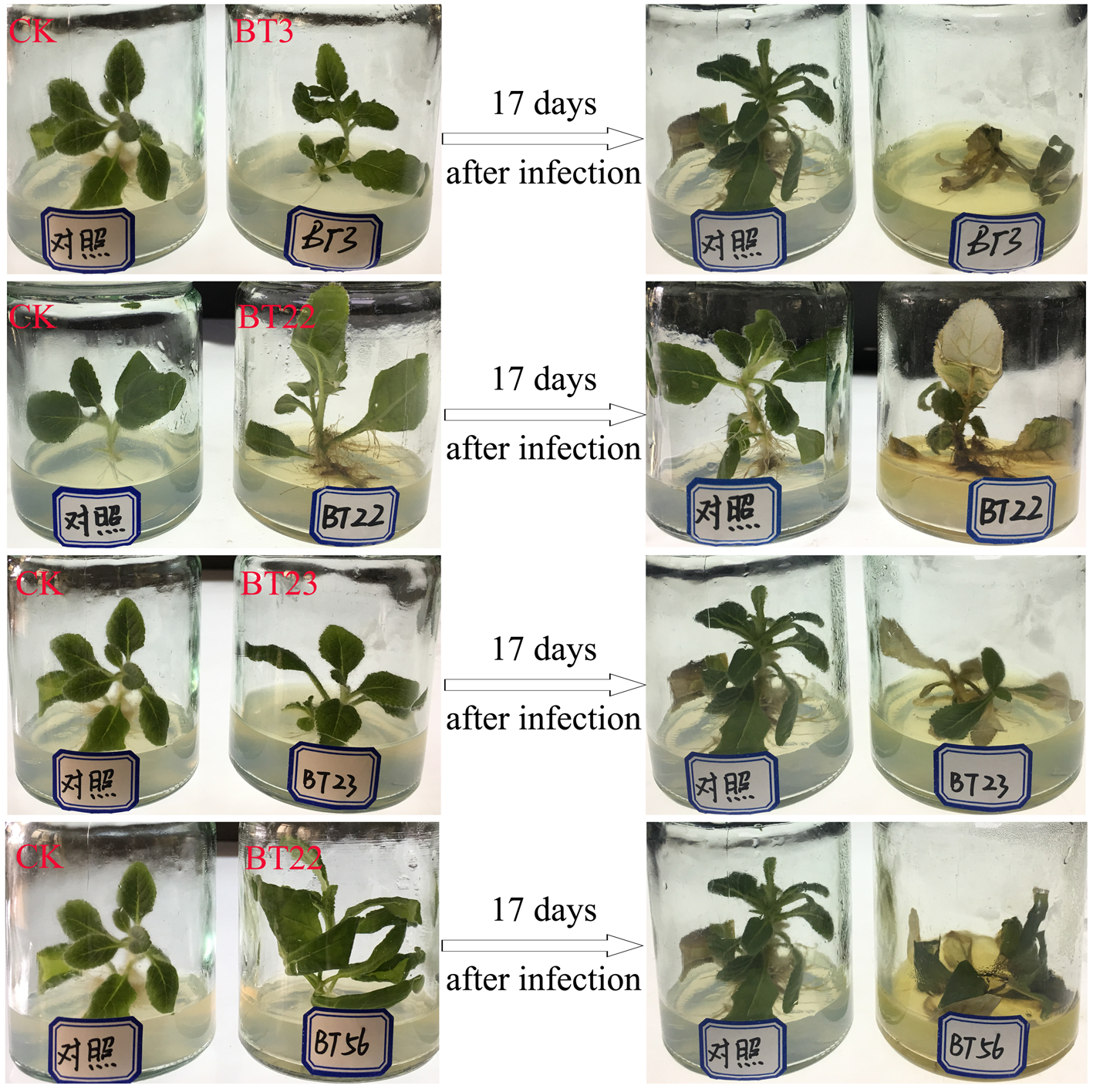


**Supplementary FIGURE S4 | Assessment of the pathogenicity of specific QS bacteria isolated from the diseased soil.** CK: Equal amount of LB broth medium was used as a negative control (CK). The prefixes “BT” indicate the QS bacteria isolated from the diseased soil.


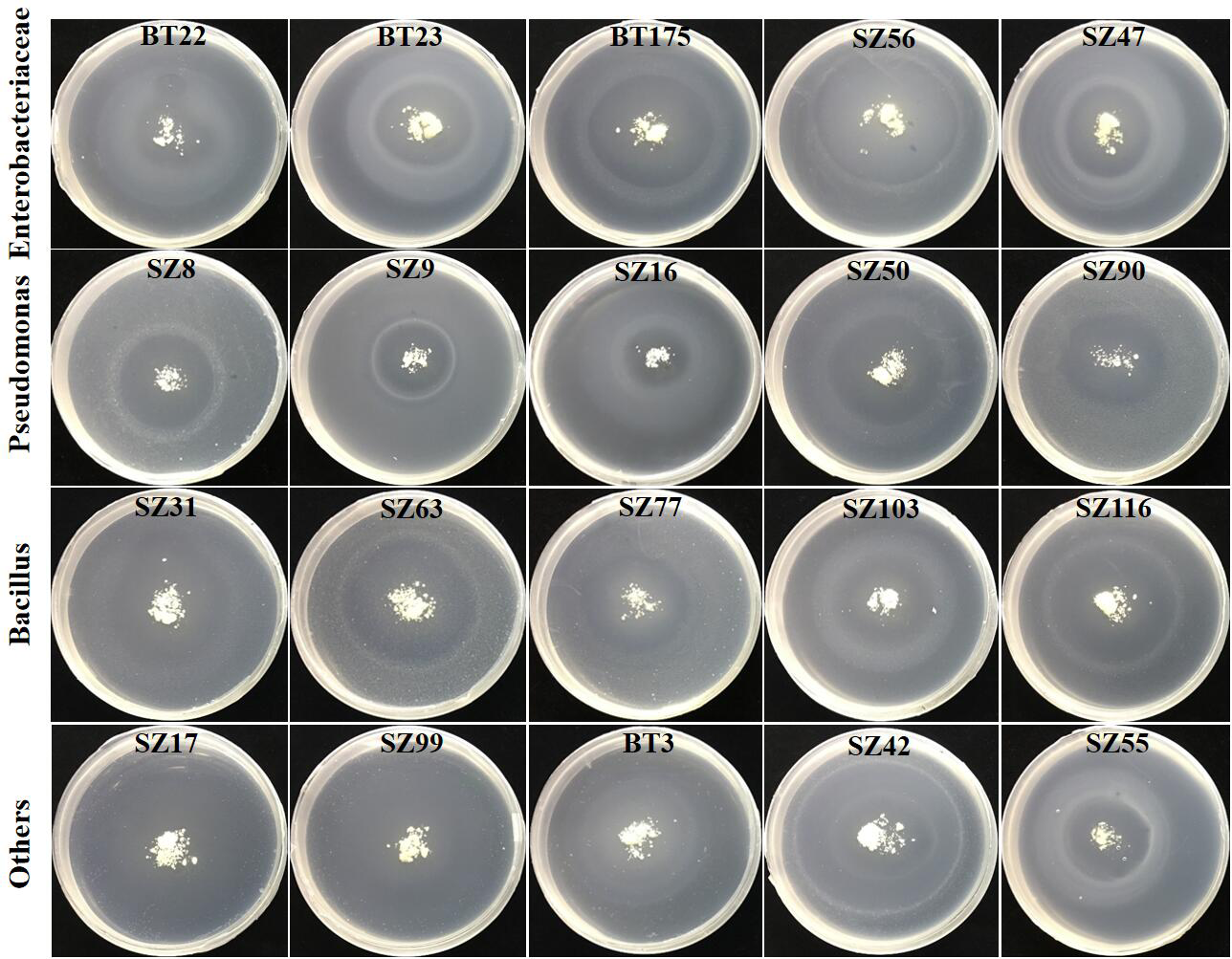


**Supplementary FIGURE S5 | Assessment of the chemotaxis of specific QS bacteria isolated from the *R. glutinosa* rhizosphere soil by drop assay.** The prefixes “SZ” and “BT” indicate the QS bacteria isolated from the NP soil and the BT soil, respectively. The name before the petri dishes indicated the taxonomic classification of QS bacteria in the same row. More details about the molecular identification of QS bacteria were shown in Supplementary Figures S2 and S3.


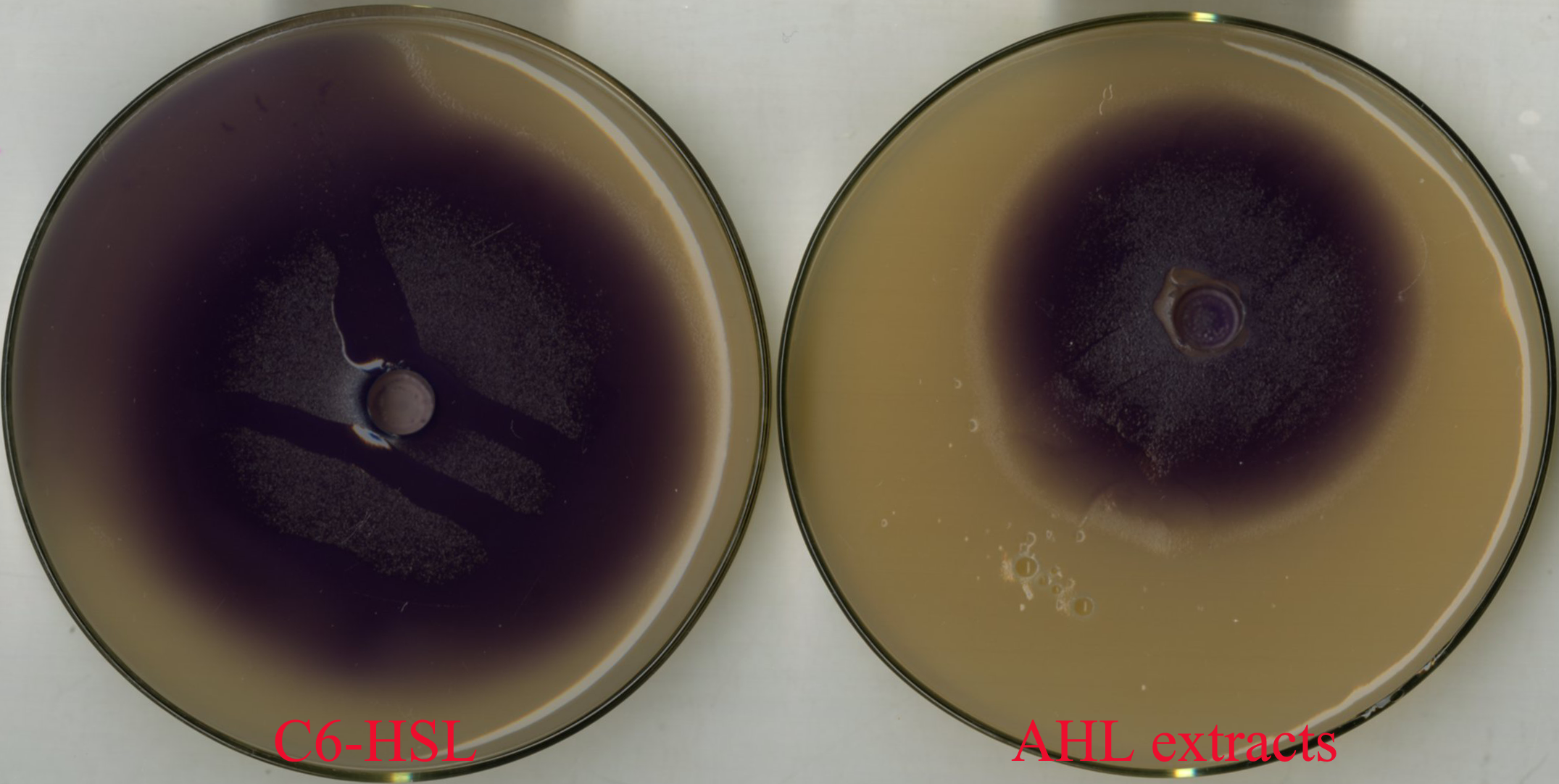


**Supplementary FIGURE S6 | Detection of QS signal molecules (AHLs) extracted from the culture broth of *Pseudomonas brassicacearum* SZ50 by biosensor CV026 through well-diffusion assay.** The left petri dish containing 6 μL of 10 μmol·mL^-1^ N-hexanoyl-l-homoserine lactone (C6-HSL) in the well was used as a positive control.


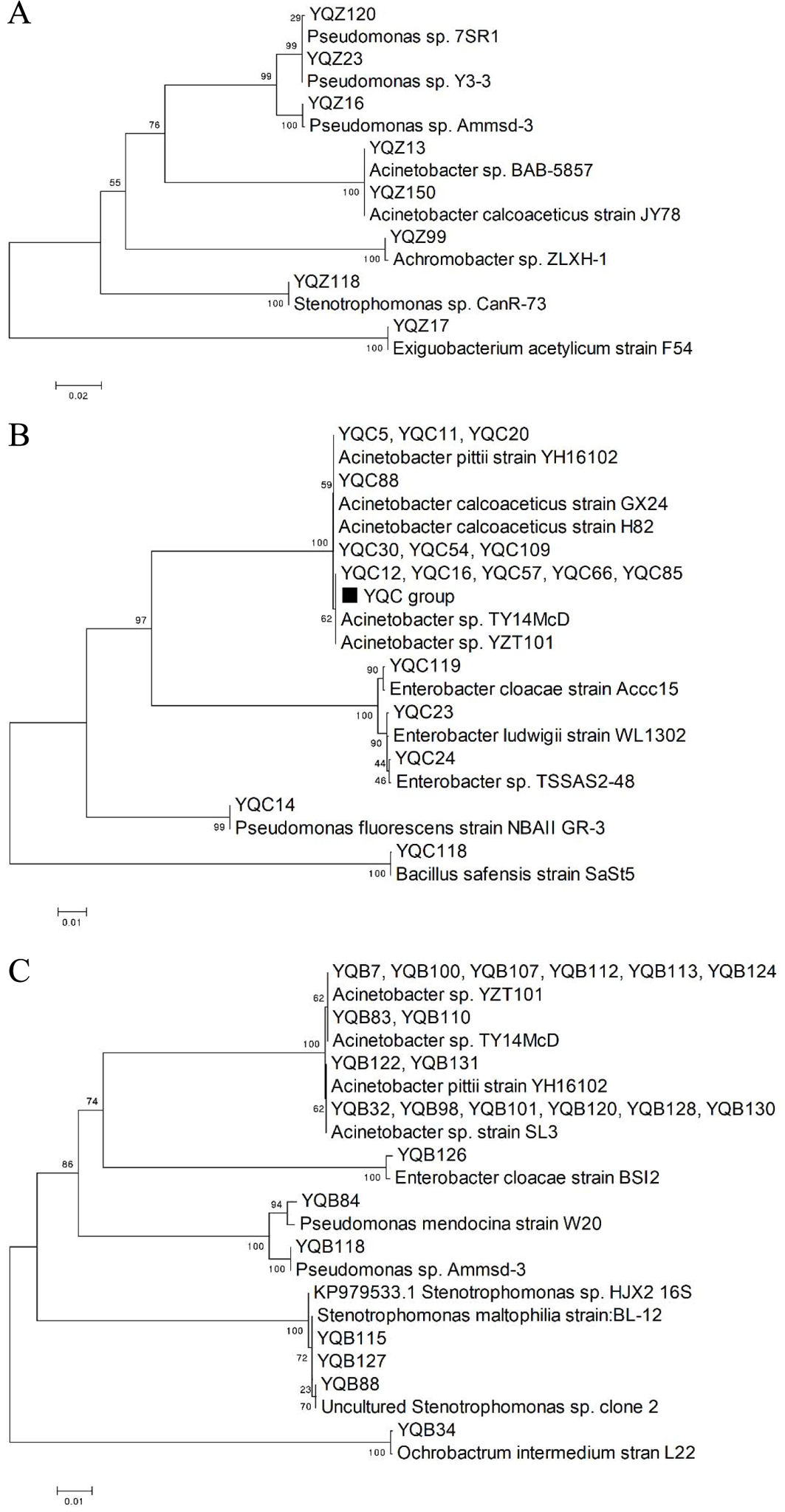


**Supplementary FIGURE S7 | The phylogenetic trees of 16S rDNA genes of QQ bacteria isolated from the newly planted soil (NP), the two-year consecutively cropped soil (CC) and the diseased soil (BT).** The prefixes “YQZ”, “YQC” and “YQB” indicate the QS bacteria isolated from the NP soil, the CC soil and the BT soil, respectively. B: YQC group includes strains YQC15, YQC18, YQC33, YQC34, YQC41, YQC42, YQC43, YQC44, YQC45, YQC47, YQC48, YQC49, YQC50, YQC60, YQC61, YQC68, YQC74, YQC75, YQC79, YQC84, YQC117 and YQC120.


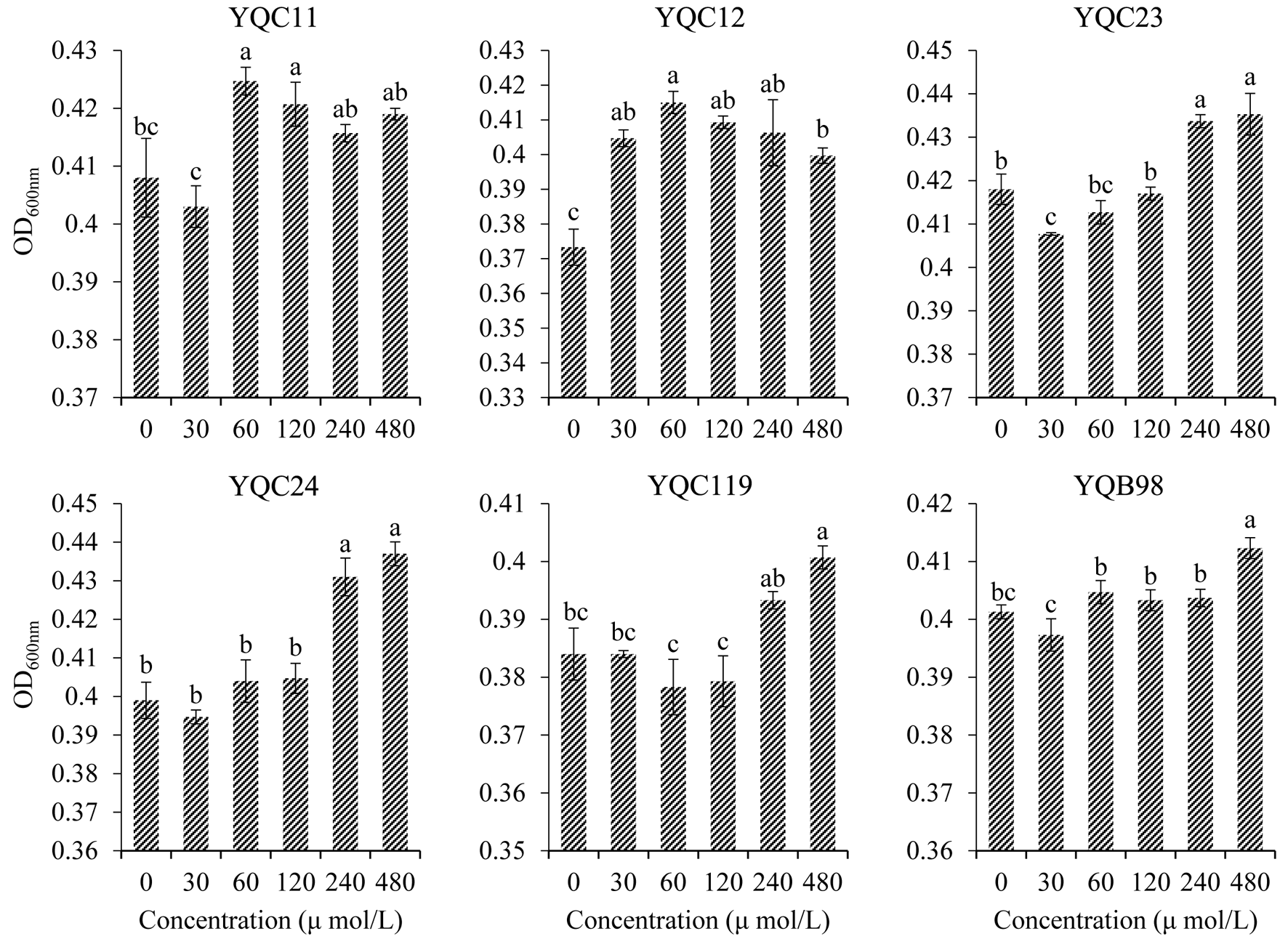


**Supplementary FIGURE S8 | Effects of the phenolic acid mixture on the growth of specific QQ bacteria isolated from the two-year consecutively cropped soil (CC) and the diseased soil (BT) of *R. glutinosa*.** The prefixes “YQC” and “YQB” indicate the QQ bacteria isolated from the CC soil and the BT soil, respectively. Data are presented as means ± standard errors.


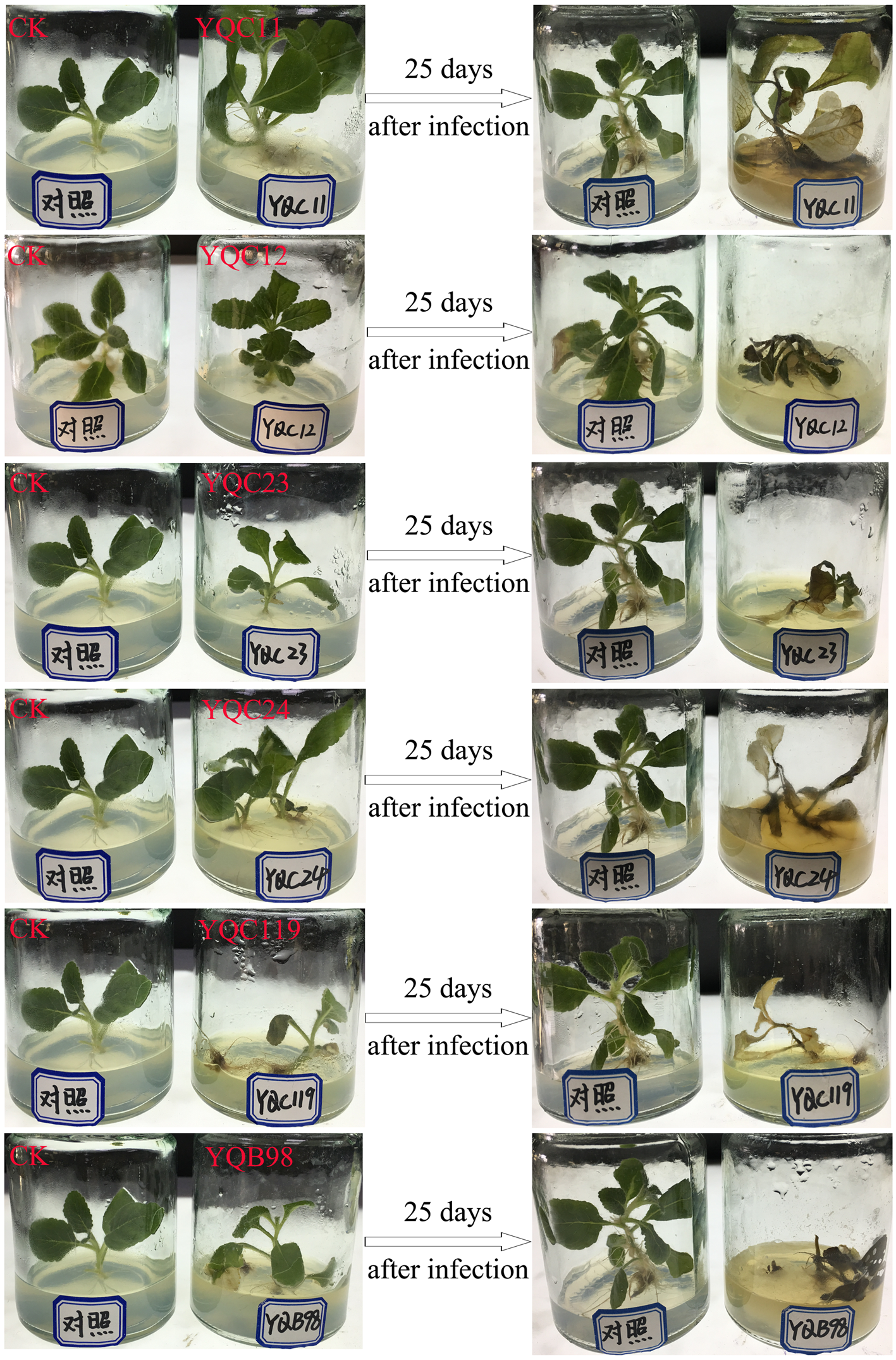


**Supplementary FIGURE S9 | Assessment of the pathogenicity of specific QQ bacteria isolated from the two-year consecutively cropped soil (CC) and the diseased soil (BT).** CK: Equal amount of LB broth medium was used as a negative control (CK). The prefixes “YQC” and “YQB” indicate the QQ bacteria isolated from the CC soil and the BT soil, respectively.

**Supplementary TABLE S1 | Antagonistic activity assessment of QS bacteria isolated from the newly planted soil and the diseased soil.**

| Isolate code | Against AF^#^ | Isolate code | Against AF | Isolate code | Against FOX^$^ |
| --- | --- | --- | --- | --- | --- |
| SZ1 | 61.5%^*^ | SZ86 | 65.00% | SZ16 | 39.70% |
| SZ4 | 62.50% | SZ88 | 62.50% | SZ88 | 42.90% |
| SZ9 | 63.80% | SZ90 | 62.50% | SZ90 | 40.00% |
| SZ14 | 60.00% | SZ92 | 57.50% | SZ92 | 40.00% |
| SZ16 | 57.50% | SZ93 | 60.00% | SZ93 | 45.70% |
| SZ17 | 65.00% | SZ95 | 65.00% | SZ95 | 42.90% |
| SZ18 | 67.50% | SZ99 | 65.00% | BT3 | 48.00% |
| SZ21 | 63.80% | SZ107 | 55.00% | BT13 | 64.00% |
| SZ22 | 70.00% | SZ109 | 60.00% | BT22 | 56.00% |
| SZ23 | 60.00% | SZ116 | 70.00% | BT25 | 76.00% |
| SZ25 | 62.50% | SZ117 | 61.30% | BT26 | 60.00% |
| SZ27 | 50.00% | SZ123 | 60.00% | BT33 | 56.00% |
| SZ28 | 67.50% | SZ126 | 60.00% | BT51 | 72.00% |
| SZ34 | 65.00% | SZ143 | 65.00% | BT64 | 60.00% |
| SZ50 | 65.00% | BT7 | 65.00% | BT83 | 28.60% |
| SZ80 | 62.50% |  |  | BT84 | 58.00% |
| SZ82 | 65.00% |  |  | BT104 | 54.00% |
| SZ83 | 63.80% |  |  | BT117 | 54.00% |
| SZ84 | 63.80% |  |  | BT118 | 54.00% |

The prefixes ‘SZ’ and ‘BT’ mean the QS bacteria isolated from the newly planted (NP) soil and the diseased (BT) soil, respectively. ^#^Antagonistic activity against *Aspergillus flavus* (AF). ^$^Antagonistic activity against *Fusarium oxysporum* (FOX). ^*^The antagonistic activity of QS bacteria against fungal pathogens were calculated according to the following formula: (R_ck_-R_tr_)/R_ck_. R_tr_ represents the mycelial radius of a fungal pathogen that was co-cultured with the isolated antagonistic bacteria. R_ck_ represents the mycelial radius of a fungal pathogen without co-culture with the isolated antagonistic bacteria.
